# Natural Compounds in Non-Melanoma Skin Cancer: Prevention and Treatment

**DOI:** 10.3390/molecules29030728

**Published:** 2024-02-04

**Authors:** Szymon Kowalski, Julia Karska, Maciej Tota, Katarzyna Skinderowicz, Julita Kulbacka, Małgorzata Drąg-Zalesińska

**Affiliations:** 1 Faculty of Medicine, Wroclaw Medical University, Pasteura 1, 50-367 Wroclaw, Poland; szymon.kowalski@student.umw.edu.pl (S.K.); maciej.tota@student.umw.edu.pl (M.T.); katarzyna.skinderowicz@student.umw.edu.pl (K.S.); 2 Department of Psychiatry, Wroclaw Medical University, Pasteura 10, 50-367 Wroclaw, Poland; julia.karska@student.umw.edu.pl; 3 Department of Molecular and Cellular Biology, Faculty of Pharmacy, Wroclaw Medical University, Borowska 211A, 50-556 Wroclaw, Poland; 4 Department of Immunology and Bioelectrochemistry, State Research Institute Centre for Innovative Medicine, Santariškių 5, 08410 Vilnius, Lithuania; 5 Department of Human Morphology and Embryology, Division of Histology and Embryology, Faculty of Medicine, Wroclaw Medical University, T. Chalubińskiego 6a, 50-368 Wroclaw, Poland; malgorzata.drag-zalesinska@umw.edu.pl

**Keywords:** natural compounds, natural agents, non-melanoma skin cancer, NMSC

## Abstract

The elevated occurrence of non-melanoma skin cancer (NMSC) and the adverse effects associated with available treatments adversely impact the quality of life in multiple dimensions. In connection with this, there is a necessity for alternative approaches characterized by increased tolerance and lower side effects. Natural compounds could be employed due to their safety profile and effectiveness for inflammatory and neoplastic skin diseases. These anti-cancer drugs are often derived from natural sources such as marine, zoonotic, and botanical origins. Natural compounds should exhibit anti-carcinogenic actions through various pathways, influencing apoptosis potentiation, cell proliferation inhibition, and metastasis suppression. This review provides an overview of natural compounds used in cancer chemotherapies, chemoprevention, and promotion of skin regeneration, including polyphenolic compounds, flavonoids, vitamins, alkaloids, terpenoids, isothiocyanates, cannabinoids, carotenoids, and ceramides.

## 1. Introduction

Cutaneous malignancies represent the most prevalent category of cancers diagnosed worldwide, with an estimated 1.5 million new cases projected in 2020. Within this estimate, approximately 325,000 new incidences of melanoma and 1.2 million non-melanoma skin cancers (NMSC) were reported by the International Agency for Research on Cancer of the World Health Organization (WHO). The death rates reached then 64,000 and 57,000, respectively. Notably, in most regions worldwide, skin cancer manifests more frequently in men than in women, according to the WHO. 

Both melanoma and NMSC are complex conditions, mainly attributed to prolonged and unprotected exposure to natural ultraviolet (UV) rays or UV lamps. The predominant deleterious effects of UV are mediated primarily by oxidative stress, which disrupts signal transduction pathways such as nuclear factor-kappa beta (NF-κB)/p65, mitogen-activated protein kinase (MAPK), Janus kinase (JAK), signal transduction and activation of transcription (STAT), and nuclear factor erythroid 2-related factor 2 (Nrf2). These alterations damage certain biomolecules and compromise the integrity of skin cells, resulting in skin damage [1]. While UV radiation is the primary instigator of skin cancer, other contributing factors encompass immunosuppression, viruses, mutagens present in food, mutagenic chemicals, and genetic predisposition [2,3].

NMSCs encompass basal cell carcinoma (BCC), squamous cell carcinoma (SCC), and actinic keratosis (AK). Among these, BCC predominates as the most common carcinoma among Caucasians, with an estimated BCC/SCC ratio of approximately 2.5:1 in the general population [4]. The metastatic potential of basal cell carcinoma (BCC) is less than 0.1%, while that of squamous cell carcinoma (SCC) ranges from 0.3% to 3.7% [5]. 

BCC commonly manifests after the age of 50 and usually appears in sun-exposed areas of the body, including the scalp, face, and forehead. BCC exhibits various clinical types, with the nodular type being the most frequently observed variant [6]. BCCs are tumors driven by the Hedgehog (Hh) pathway, exhibiting similarities to basal keratinocytes in the follicular and interfollicular epidermis. This resemblance suggests their likely origin from these specific cells [7]. Mutations in Hh pathway genes, particularly PTCH and SMO, are prevalent in BCC. Somatic PTCH mutations are found in 90% of sporadic BCCs, and gain-of-function mutations occur in SMO [8]. Additionally, the phosphatidylinositol-3-kinase (PI3K) pathway promotes Hh signaling in oncogenesis. Downstream components, including S6-kinase 1 (S6K1) and atypical protein kinase C (aPKC), contribute to this process. The Hh target gene, aPKC, phosphorylates Gli1, activating its DNA binding and initiating positive feedback that amplifies Gli-dependent transcription in BCC [9].

SCC arises from the malignant proliferation of epidermal keratinocytes [10]. Typically found in sun-exposed areas, SCC can manifest anywhere in the body and may develop de novo or from a predisposing lesion, including AK [11]. SCC manifests through intricate molecular alterations. Frequent TP53 mutations and p16INK4a inactivation drive uncontrolled cell growth [12,13]. Dysregulated EGFR and RAS pathways contribute to aberrant cellular processes [14,15]. Notch signaling disruption, UV-induced DNA damage, and cyclin D1 overexpression are additional molecular features in SCC oncogenesis [13,16,17]. The inactivation of tumor suppressor genes, including PTEN, further propels uncontrolled cellular proliferation [18]. The pathogenesis of SCC involves aberrant DNA methylation, histone modifications, and dysregulation of long non-coding RNAs or microRNAs as part of epigenetic changes [18,19,20,21].

AK are neoplasms derived from keratinocytes, arising on skin exposed to chronic UV radiation [22]. Prevalent in older individuals with light pigmentation, their occurrence ranges from 11% to 60% in non-Hispanic whites over 40 years old [23]. Pathogenesis involves alterations in pathways that regulate cell growth, differentiation, inflammation, and immunosuppression due to UV radiation, tissue remodeling, oxidative stress, and impaired apoptosis [24,25]. Traditionally perceived as a distinct pre-malignant lesion, recent evidence suggests that AK are part of a disease continuum existing between subclinical photodamaged skin and SCC [26]. Molecular analyses have revealed a shared genetic profile between AK and SCC, which includes alterations in p53, p16INK4a, MYC, and the epidermal growth factor receptor [26,27,28]. Moreover, two distinct molecular profiles have recently been distinguished in AK: “lesional AK”, with a profile akin to SCCs, highlighting the crucial role of the VEGF pathway in their development, and “non-lesional AK”, with a profile resembling normal skin tissue (Table 1) [29].

Considering the morbidity and fatality rates associated with NMSCs, emphasis is placed on the development of effective preventive and treatment strategies. Given the involvement of ultraviolet radiation in their pathogenesis, photoprotection is essential for the prevention of non-melanoma skin cancers [35]. Additional preventive measures encompass the early identification of high-risk individuals and the use of agents such as retinoids, known for their efficacy in reducing the risk of pre-malignant cells progressing into carcinomas [36]. 

Current approaches for treating skin cancer include surgical procedures, radiation therapy, lasers, phototherapy, chemotherapy, immunotherapy, and cryosurgery [37,38,39]. In the context of topical chemotherapies, 5-fluorouracil, imiquimod, and tretinoin find application in addressing NMSCs [39,40]. 

Nonetheless, chemotherapeutic agents face notable challenges, encompassing substantial adverse effects and the emergence of multidrug resistance mechanisms, such as efflux systems, target amplification, and modifications in drug kinetics [41]. To overcome these challenges, various strategies, such as the use of nanoparticles, liposomes, and micellar drug delivery systems, have been explored [42,43]. 

Here, we examine recent research on using natural compounds in addressing NMSC from the past 10 years. Given the limitations of current NMSC treatments, this paper explores novel natural substance-based strategies, highlighted by their documented efficacy in the literature. This review not only discusses the potential of phytochemical agents from plant extracts, known for their anti-cancer properties, but also addresses the challenges in their application, such as variability in preparation and dosage. The aim is to provide a comprehensive overview of the latest developments in natural compounds for NMSC prevention and treatment.

## 2. Conventional Treatment

### 2.1. 5-Fluorouracil

Topical 5-fluorouracil (5-FU) has been described as a treatment for malignant and non-malignant skin conditions [44,45]. Acting as an agent, 5-FU enters cells using the same mechanism as uracil. The metabolism of 5-fluorouracil (5-FU) or its derivatives disrupts intracellular nucleotide pools. This disruption leads to the incorporation of false bases, specifically 5-FU, an analog of uracil, into the DNA. Consequently, this alteration interferes with the processing and functioning of RNA, ultimately resulting in DNA damage. The cytotoxic effects of 5-FU manifest primarily in rapidly proliferating cells within abnormal skin [46,47]. Following the topical application of 5-FU, there is typically a progression of inflammation, erosion, and disappearance of the abnormal lesions. The selective cytotoxicity of 5-FU, with minimal impact on normal skin cells, positions it as a promising therapeutic option in dermato-oncology [47].

### 2.2. Photodynamic Therapy

Photodynamic therapy (PDT) is a rapidly advancing non-invasive treatment with notable advantages over alternatives [48]. It uses a tumor-targeting photosensitizer, potentially requiring metabolic synthesis, activated by specific-wavelength light. The mechanisms of PDT involve the generation of singlet oxygen (^1^O_2_) through photosensitizer excitation, inducing necrotic, autophagic, or apoptotic tumor cell destruction [49,50]. PDT is recognized for its therapeutic effectiveness in treating certain types of cancers, including non-melanoma skin cancers. Successful in treating BCC, Bowen’s disease, and AK, PDT offers advantages such as reduced pain and improved patient tolerance, achieving excellent cosmesis. Variable outcomes are observed for nodular BCC, with enhanced outcomes after pre-treatment and repeated PDT cycles. Aggressive BCC subtypes and invasive SCC are less suitable for PDT. Emerging developments involve preventing “field cancerization”, which refers to molecular changes stemming from p53 tumor suppressor gene mutations and leading to subclinical malignant potential after conventional treatment [51]. The significance of cancer-associated fibroblasts and macrophages in resistance to PDT for NMSCs is increasingly acknowledged [52,53].

### 2.3. Laser Therapy

Laser therapy is a treatment that involves applying a focused light source of a specific wavelength to the tumor. Lasers can be used to cut, burn, or destroy tissues, which can be applied to NMSCs [42]. The treatment of skin cancer involves the use of four main types of lasers: solid-state, diode, dye, and gas lasers [54]. There are two laser treatments used especially for BCC: carbon dioxide laser and pulsed contrast laser. The main benefits of these procedures are satisfactory cosmetic results as well as high cure rates; however, histological evaluations cannot be conducted afterward. In the case of laser therapy for SCC, the literature is far more limited. Initial findings have shown encouraging outcomes in the treatment of SCCs in situ. However, the effectiveness of these treatments in managing invasive SCC has not been conclusively proven [55].

### 2.4. Cryosurgery

Cryosurgery involves the delivery of liquid nitrogen to freeze the target tumor tissue rapidly and then thaw slowly, leading to local cellular destruction. Regarding NMSC, cryosurgery may be considered in low-risk BCC and should be avoided in SCC due to possible metastasis [56]. Numerous studies have confirmed the high efficacy of cryosurgery in the treatment of BCC, with a 5-year recurrence-free rate of 95–99% [57,58,59]. There are several benefits of cryosurgery. Of those, cryosurgery is quick, cost-effective, and requires no anesthesia. Some potential adverse effects should also be acknowledged, e.g., post-treatment prolonged edema, neuropathic pain, scarring, and hypopigmentation [60].

### 2.5. Hedgehog Inhibitors

Aberrantly activated hedgehog signaling plays a pivotal role in developing cancers, including basal cell carcinoma (BCC). Patched homolog 1 (PTCH1) regulates the hedgehog pathway by binding to the smoothened (SMO) protein, an activator of this pathway. Over 90% of all BCC cases exhibit deletion mutations in the PTCH1 gene or activating mutations in the SMO gene, leading to enhanced hedgehog pathway activity and the development of BCC. In recent years, inhibitors have been developed to attenuate hedgehog signaling. Two of them, vismodegib and sonidegib, have been approved for use in advanced and metastatic BCCs. Other molecules are currently under investigation in clinical trials [61,62,63,64].

### 2.6. Immunotherapy

Immunotherapy is playing an increasingly crucial role in the treatment of advanced cancers. In the case of skin cancers, it is employed in locally advanced and unresectable cSCCs (cutaneous Squamous Cell Carcinomas), as well as, more recently, in BCCs resistant to hedgehog inhibitors. In NMSC immunotherapy, checkpoint inhibitors are used to assist the host immune system in more effectively combatting cancer cells by modulating the immune response and enhancing the activity of cytotoxic T cells [65,66]. Effective targets for checkpoint inhibitors in NMSC include the PD-1/PD-L1 pathway (pembrolizumab, cemiplimab, and avelumab) and CTLA-4 (ipilimumab) [37]. The use of neoadjuvant immunotherapy can allow for the surgical treatment of previously unresectable diseases and potentially improve the quality of life for individuals with locally advanced diseases [67]. Despite satisfactory responses to immunotherapy in NMSCs, the associated immune-related adverse events require monitoring. Consequently, clinical research efforts should focus on discovering a novel treatment that enhances tumor response while minimizing toxicity [37].

## 3. Polyphenolic Compounds

Polyphenolic compounds are a large family of naturally occurring substances found abundantly in plants. They are known for their diverse structure and are primarily composed of phenols. Common types of polyphenols include flavonoids, phenolic acids, stilbenes, and lignans. These compounds are recognized for their antioxidant properties and are found in a variety of foods and beverages, such as fruits, vegetables, tea, wine, and chocolate. In medicine, polyphenolic compounds are recognized for their potential health benefits, largely due to their antioxidant and anti-inflammatory properties. They may contribute to the prevention and treatment of a variety of diseases, including cardiovascular diseases, cancers, and neurodegenerative disorders. The antioxidant activity of polyphenols can help combat oxidative stress, which is linked to many chronic diseases. Moreover, these compounds may play a role in modulating the immune system and influencing gut microbiota, which can have further health implications [68]. 

**Figure 1:**
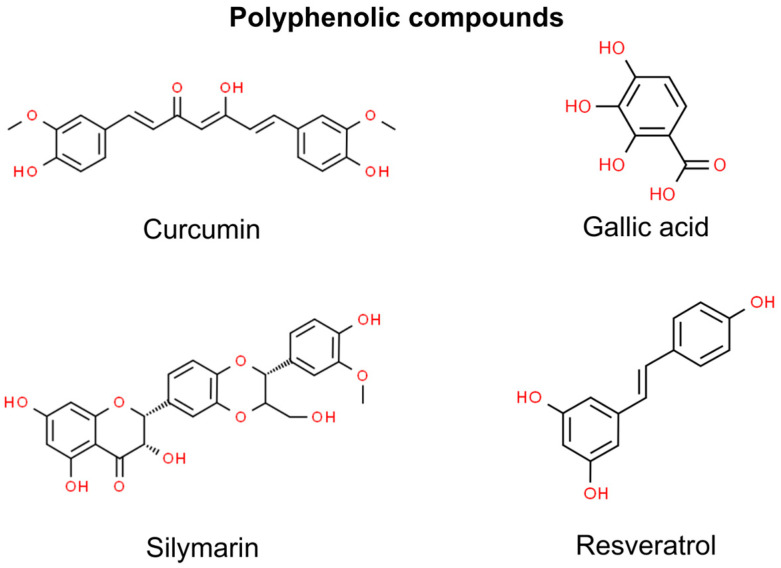
Chemical structures of selected polyphenolic compounds showing anti-NMSC effect.

To date, certain polyphenols or their mixtures have been found to have preventive or therapeutical effects on non-melanoma cancers—curcumin, gallic acid, silymarin, resveratrol, cinnamic acid, and rosmarinic acid. However, recent scientific studies have indicated that cinnamic acid and rosmarinic acid exhibit therapeutic action against head and neck SCC rather than skin SCC (Figure 1) [69,70,71].

### 3.1. Curcumin

Curcumin, a vibrant yellow plant polyphenol, has played a multifaceted role throughout history, serving as both a cherished spice and a valuable medicinal agent. It is a major part (74.9%) of a spice, curcuminoid, which likewise consists of desmethoxycurcumin (DMC) (20.1%) and bisdemethoxycurcumin (BDMC) (4.9%) [72]. Derived primarily from *Curcuma longa*, more commonly known as turmeric, curcuminoids are meticulously isolated from the rhizomes of this plant [73]. Curcumin’s rich history encompasses diverse applications, ranging from its traditional use as an herbal supplement to its incorporation into food coloring and preservation, flavoring, and cosmetics [74]. 

Notwithstanding its lack of official approval for therapeutic use, curcumin has demonstrated efficacy against diverse diseases, including breast, ovarian, and prostate cancer, human immunodeficiency virus, epilepsy, psoriasis, and diabetes. Notably, curcumin exhibits a favorable safety profile even at gram doses, without side effects up to 8000 mg per day [75,76,77,78]. Researchers have explored the potential application of curcumin as a pharmaceutical agent to manage and treat cancer-related symptoms, such as pain, fatigue, depression, and neurodegeneration, yielding promising results [79]. Curcumin exerts its impact on cancer progression through the selective modulation of various signaling pathways [80].

In terms of UVB exposure causing damage both in vitro and in animals, topical application of curcumin significantly mitigates acute UVB-induced damage by reducing the release of lactate dehydrogenase, intracellular ROS, and DNA damage [81]. An animal study showed that both superficial and oral applications of curcumin before chronic UV exposure delayed tumor onset [82]. 

UVB exposure triggers mTOR and fibroblast growth factor receptors (FGFR) signaling pathways that play a pivotal role in skin tumorigenesis, including SCC, BCC, and AK. Notably, the pre-treatment application of the C3 curcuminoid complex hinders UVB-induced fibroblast growth factor-2 (FGF-2) expression, FGF-2-mediated cell proliferation, progression, and colony formation, along with suppressing mechanistic target of rapamycin (mTOR) pathway (mTORC1/mTORC2 network) as well as FGFR2 phosphorylation in JB6 epithelial cells sensitive to promotion. Oral application of the C3 complex on mice significantly inhibits UVB-induced epidermal hyperplasia and hyperproliferation likewise [83]. 

The predominant body of research on curcumin with respect to non-melanoma primarily focuses on SCC, particularly within the domain of head and neck SCC (HNSCC) [84]. The investigation into the involvement of curcumin in HNSCC was carried out through a clinical trial. It demonstrated that the administration of microgranular curcumin might be linked to a notable reduction in FGF-2, macrophage colony-stimulating factor, and interleukin-17 involved in the modulation of angiogenesis and cellular invasion of HNSCC. These findings suggest that curcumin could serve as a prospective angiogenic inhibitor in HNSCC, potentially impeding the advancement of pre-neoplastic lesions into invasive cancer [85]. 

Curcumin’s hydrophobic nature and low solubility limit its oral bioavailability, posing challenges for therapeutic use. Topical treatment protocols may mitigate these challenges and offer clinical advantages. Current research has explored nanoformulations, such as diverse types of liposomes or PEGylated solid lipid nanoparticles, to enhance solubility and address these issues [86]. The topical application of a curcumin-loaded liposome-siRNA complex induced a more pronounced inhibition of SCC cell model growth and apoptosis events compared with the control group [87]. Moreover, the curcumin-loaded nanopatterned films presented good cytotoxicity against the same SCC cell model [88]. However, topical curcumin treatment may be restricted by the potential for contact dermatitis, a phenomenon observed in humans [89]. The clinical trial mentioned above presented a resolution to the challenges associated with the oral administration of curcumin. The oral transmucosal delivery of microgranular curcumin has demonstrated heightened curcumin bioavailability, aligning with significant biological effects that have the potential to impede the progression of pre-neoplastic lesions to invasive SCC [85]. Notably, piperine, a natural anti-cancer agent, enhances curcumin bioavailability by 2000% by inhibiting glucuronidation [90]. Nevertheless, ongoing advances in the curcumin formulation may further optimize its responses and efficacy.

### 3.2. Gallic Acid

Gallic acid (GA) is a naturally occurring compound found in various plants, including gallnuts, sumac, witch hazel, tea leaves, oak bark, and others. It is known for its potent antioxidant properties. GA is often used in the pharmaceutical and food industries due to its health benefits and as a component in some inks and dyes. It has been studied for its potential therapeutic effects, including an anti-cancer effect [91].

GA acts against BCC by targeting and inhibiting HSP90AB1, a protein associated with cancer aggressiveness. GA reduces the migration and proliferation of BCC cells without increasing Reactive Oxygen Species (ROS) levels or causing death in keratinocytes (healthy skin cells). This suggests that GA can effectively target and impair BCC cells while preserving healthy cells, making it a potential therapeutic agent for treating BCC [92].

GA impacts SCC by inhibiting HSP90AB1, a protein associated with cancer’s invasiveness and aggressiveness. GA effectively reduces cell migration and proliferation in SCC, promoting cell death and significantly lowering HSP90AB1 levels in SCC cells [92]. This action of GA highlights its potential as a therapeutic agent in treating SCC by targeting specific cancer-related proteins and pathways.

### 3.3. Silymarin

Silymarin is derived from the seeds of milk thistle (*Silybum marianum*), which is an annual Mediterranean native herb [93]. This extract is a flavonolignan complex and contains other polyphenolic compounds, amongst which silibinin can be found as the major one [94]. This compound has built itself a positive reputation for several centuries due to its hepatoprotective effects regarding its antioxidant and anti-inflammatory activities [95]. Lately, silymarin has also been used in dermatology and cosmetology due to its protective and preventive potential against UVB-induced NMSC in pre-clinical skin cancer studies [96]. Over the last two decades, many studies have shown its anti-cancer activity, amongst which research conducted by Agarwal and colleagues on 7, 12-Dimethylbenz[a]anthracene (DMBA)/12-O-tetradecanoylphorbol-13-acetate (TPA)-induced mouse skin carcinogenesis model indicated it. By inhibiting the activity of epidermal ornithine decarboxylase, silymarin suppressed the skin cancer progression [96]. Moreover, silibinin has been proven in vitro in animal models to act against UVB-induced thymine dimer formation, inhibiting carcinogenesis by increasing p53 levels. It also promotes DNA repair and induces anti-inflammatory responses, as well as targeting aberrant signaling pathways. Silibinin could be proven to have a successful therapeutic effect against skin SCC and BCC in humans as it targets inflammatory and oxidative stress signaling [96].

### 3.4. Resveratrol

**Figure 2:**
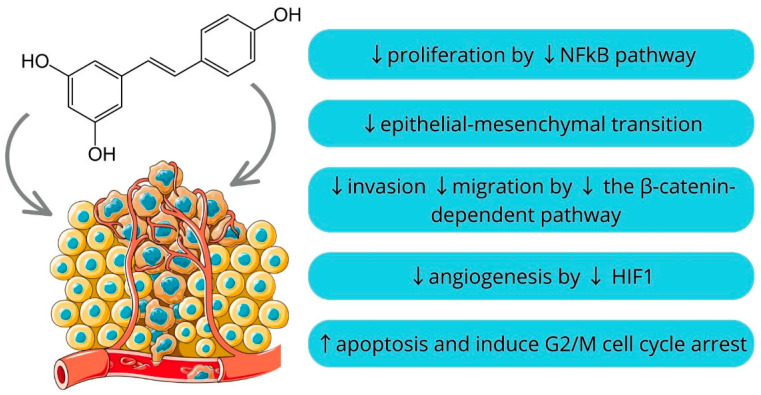
Mechanisms of resveratrol action [97,98,99,100,101,102,103,104,105,106] (parts of the figure were drawn by using pictures from Servier Medical Art).

Resveratrol is a naturally occurring polyphenol found in various plants, including grapes, berries, and peanuts. It is particularly noted for its presence in red wine. It has the potential to positively impact both the prevention and management of inflammatory disorders and cancer as well as slowing the aging process [97,98,99]. It is comprised of a pair of phenolic rings connected by a double styrene bond, creating the structure known as 3,5,4′-Trihydroxystilbene [100]. Earlier research illustrated that resveratrol has pleiotropic effects instead of depending on a single mode of action. It has been shown that resveratrol inhibits proliferation by downregulating NFkB, inhibits epithelial-mesenchymal transition dependent on TGF β/Smad, decreases the β-catenin-dependent pathway to impede invasion and migration, and influences angiogenesis by inhibiting HIF-1 and expediting its ubiquitination [101,102,103,104]. Moreover, an oral squamous cell carcinoma cell lines study showed that resveratrol induces apoptosis and G2/M phase cell cycle arrest [105]. However, determining the optimal dosage of resveratrol for maximizing its health benefits while mitigating the risk of toxicity represents a significant and ongoing area of scientific inquiry [106]. Nonetheless, resveratrol’s capacity to target various aspects of tumor development has enhanced its value as a supplementary agent in combination with other treatments, enhancing its efficacy through synergy (Figure 2) [98].

#### Resveratrol and Ursolic Acid

In the case of NMSCs, recent studies on cell lines and mice investigated the combination of resveratrol with ursolic acid. Ursolic acid is a natural triterpene pentacyclic triterpenoid compound present in various plants, such as apples and rosemary. The increasing interest in UA stems from its advantageous effects, which encompass anti-inflammatory, antioxidant, anti-apoptotic, and anti-carcinogenic properties [107,108]. In research conducted under in vivo conditions, ursolic acid, resveratrol, and the ursolic acid + resveratrol combination were topically administered before 12-O-tetracanoylphorbol-13-acetate (TPA) treatment on mouse skin to investigate their impact on TPA-induced signaling pathways, epidermal hyperproliferation, skin inflammation, inflammatory gene expression, and the promotion of skin tumors. The administration of ursolic acid + resveratrol during the promotion of skin tumors with TPA resulted in more significant inhibition of tumor multiplicity and tumor size compared with using either agent separately [109]. Therefore, resveratrol and ursolic acid interact synergistically. It has been suggested that resveratrol may enhance the effects of ursolic by preventing its metabolism and, consequently, maintaining its intracellular concentration [110].

## 4. Flavonoids

**Figure 3:**
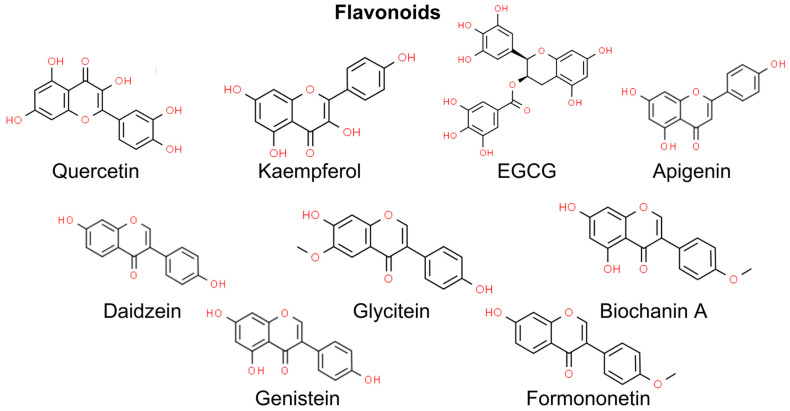
Chemical structures of selected flavonoids showing an anti-NMSC effect. EGCG—epigallocatechin gallate.

Flavonoids are phytochemical compounds classified into seven subclasses, namely flavonols, flavones, isoflavones, anthocyanidins, flavanones, flavanols, and chalcones [111]. Flavonoids are well-established antioxidants since they scavenge ROS via inhibiting superoxide anion-producing oxidases, chelating trace metals, and activating antioxidant enzymes. Due to their antioxidant activity, flavonoids may protect from UV-induced skin damage and NMSCs. Conversely, flavonoids may also trigger excessive oxidative stress and induce apoptosis in several cancer cell types, e.g., breast cancer and colorectal cancer cell lines [112]. However, there is limited evidence of the prooxidant activity of flavonoids in the prevention and management of NMSCs. Thus, in this chapter, we focus on the antioxidant activity of flavonoids in the prevention of skin damage (Figure 3).

### 4.1. Quercetin

Quercetin (3,5,7,3′,4′-pentahydroxyflavone) shows a wide range of potential activities, including anti-cancer, anti-diabetic, anti-inflammatory, antioxidant, anti-microbial, anti-arthritic, cardioprotective, and wound-healing effects [113]. Quercetin is found in onions, grapes, berries, cherries, broccoli, and citrus fruits [114]. An in vitro study by Stevanto et al. showed a mathematically calculated Sun Protection Factor (SPF) of selected natural compounds. Despite displaying relatively low UVB protection (SPF~10) compared with apigenin (SPF = 28.8) and kaempferol (SPF = 24.9), quercetin was characterized by absorption at relatively high absorption levels (360–375 nm) compared with other flavonoids. Furthermore, quercetin showed the highest UVA protection among flavonoids, measured by the UVA/UVB ratio parameter [115].

However, quercetin is characterized by rapid metabolism and excretion, low stability, poor water solubility, and poor absorption. Thus, there is a need to elaborate an effective drug-delivery system for topical application. Nan et al. found that encasing quercetin in chitosan nanoparticles provided enhanced stability and minimal cytotoxicity while being readily absorbed by HaCaT cells and capable of passing through the epidermis layer. The authors found that quercetin blocked the NF-Κβ/COX-2 signaling pathway [116]. In another study conducted in vivo on male Wistar rats and in vitro on skin cancer A431 cell lines, Chitkara et al. presented quercetin nanoemulgel prepared using the ultrasonication emulsification method. The nanoemulgel effectively mitigated the skin edema from UVB radiation and showed neither skin irritation nor organ toxicity in male Wistar rats [117].

### 4.2. Kaempferol

Kaempferol (5,7,4′-trihydroxyflavone) is rich in tea, broccoli, cabbage, kale, beans, endives, leek, tomatoes, strawberries, and grapes. Its pharmacological activities include antioxidant, anti-inflammatory, anti-microbial, anti-cancer, cardioprotective, neuroprotective, anti-diabetic, anti-osteoporotic, anxiolytic, analgesic, and anti-allergic activities [118].

Similar to quercetin, kaempferol exhibits absorption at relatively high absorption levels (360–375 nm) compared with other flavonoids. Moreover, kaempferol retains moderate UVB protection (SPF = 24.9) [115]. Yao et al. demonstrated chemopreventive properties against UV-induced carcinogenesis in a mouse model. Mice with topically administrated kaempferol presented remarkably delayed tumor growth. The authors highlighted that the inhibitory effects of kaempferol are caused by targeting RSK2 and MSK1 [119].

### 4.3. Epigallocatechin Gallate and Gallocatechin Gallate

Epigallocatechin gallate (EGCG), found primarily in green tea, has been widely reported to display antioxidative, anti-inflammatory, anti-cancer, anti-proliferative, and chemopreventive activities [120].

A study on A431 and SCC13 human skin cancer cell lines revealed that ECGC exhibits anti-proliferation potential by inactivating β-catenin signaling. Downstream targets of β-catenin signaling, including MMPs, c-Myc, and VEGF, were also suppressed. Concomitantly, EGCG decreased the levels of COX-2 and PGE2. [121]. Moreover, a study on human skin fibroblasts (HSF) demonstrated that ECGC delayed UVA-induced photoaging [122].

Nevertheless, EGCG is unstable under ambient conditions, while its epimer, gallocatechin gallate (GCG), is chemically more stable. Sheng et al. showed that GCG may protect against UVB-induced skin damage by increasing skin elasticity and the number of collagen fibers in a study on a mouse model mouse model. Based on the findings from transmission electron microscopy (TEM), the authors suggested that GCG alleviates UVB-induced aberrations in mitochondria and the formation of melanosomes [123].

### 4.4. Apigenin

Apigenin (4′,5,7-trihydroxyflavone) is a compound of parsley, chamomile, celery, vine spinach, artichokes, and oregano investigated on anti-cancer mechanisms in SCC cell lines [124]. Wang et al. showed that apigenin might induce apoptosis by downregulating sulfiredoxin expression and activating the MAPK signaling pathway [125]. In another in vitro SCC model, apigenin administration resulted in a decrease in microtubule-associated protein 1 light chain 3 (LC3) turnover and green fluorescent protein (GFP-LC3), suggesting autophagy inhibition [126]. Stevanto et al. showed that apigenin has the greatest potential in UVB protection among flavonoids (SPF = 28.8) [115]. Furthermore, apigenin was found to suppress the expression of IKKα in mouse cell lines. As a result, epithelial-mesenchymal transition (EMT) was diminished, indicating potential anti-metastatic activity [127]. Apigenin has also been found to reduce the synthesis of COX-2, PGE2, EP1, and EP2 in mouse models [128].

### 4.5. Isoflavonoids

Daidzein, genistein, biochanin A, glycitein, and formononetin are phytoestrogen isoflavolones. Phytoestrogens are compounds derived from plants that are structurally similar to 17β-estradiol. [129,130]. Consequently, phytoestrogens may act through estrogen receptors to increase the production of collagen, hyaluronic acid, and extracellular protein matrix. Furthermore, phytoestrogens contribute to increased skin vascularization, cell proliferation, and protection against oxidative stress [131].

Daidzein (4′,7-dihydroxyisoflavone) is a phytoestrogen isoflavone derived from leguminous plants, particularly soybeans. 7,3′,4′-trihydroxyisoflavone (734-THIF), a secondary metabolite of daidzein, has been found to have antioxidant, atopic dermatitis-relieving, melanin inhibiting, and skin cancer chemopreventive activities [132,133,134]. Similar to quercetin, due to its poor water solubility and poor absorption, its topical administration is limited. Thus, Huang et al. proposed E100 (EE)–polyvinyl alcohol (PVA)-loaded 734-THIF nanoparticles that present improved water solubility and enhanced skin penetration on HaCaT cell lines [132]. In another study, polyvinylpyrrolidone K30-based 734-THIF nanoparticle powder also showed increased physiochemical properties. Additionally, 734-THIF reduced the overexpression of COX-2 and MMP-9 by downregulating MAPK pathway signaling in particulate matter-exposed HaCaT keratinocytes [135]. That suggests potential anti-inflammatory, anti-pollutant, and anti-aging properties of 734-THIF.

Studies in vitro, in vivo, and animal models have shown that genistein (4′,5,7-trihydroxyisoflavone), a soybean derivative, is another candidate to protect against UVB-induced inflammation and aging. Tang et al. found in vivo that genistein suppresses inflammatory cytokines, notably CXCL1, IL-1, MIF, and PLANH1. In animal models (dorsal skin of rats), topical-administrated genistein decreased the number of skin folds and wrinkles induced by UVB. Furthermore, a diet reach in genistein in human participants significantly reduced the severity of UVB-induced wrinkling [136]. 

Another study in an animal model by Terra et al. showed a mechanism potentially linking genistein to photoprotection. NO (nitric oxide) is a factor that contributes to the inhibition of cell proliferation after UVB exposure. The authors found that genistein has antioxidant activity preventing the reaction of H_2_O_2_ with NO, consequently reducing ONOO- formation. This promotes cell proliferation and tissue protection [137].

Biochanin A (5,7-dihydroxy-4’-methoxyisoflavone) is found in red clover, chickpeas, soybeans, and other herbs [138,139]. Lim et al. observed that biochanin A inhibits the UV-induced expression of COX2 via MLK3 inhibition in vitro [140]. Since COX2 may inhibit the proliferation of SCC cells in vivo, biochanin A is a promising putative anti-cancer compound [140]. 

## 5. Vitamins

**Figure 4:**
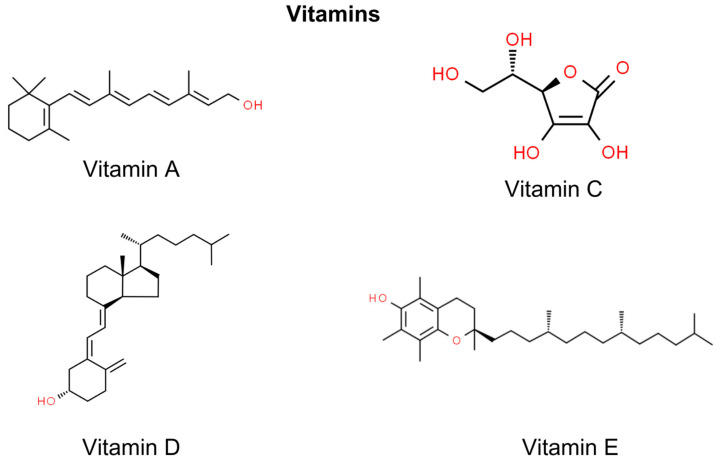
Chemical structures of selected vitamins showing anti-NMSC effect.

Vitamins have emerged as potential therapeutic agents in the prevention and treatment of NMSC, the most prevalent form of skin cancer. Retinoids, derivatives of vitamin A, are significant for their regulatory effects on keratinocyte growth, which is crucial in NMSC management [141]. Vitamin D, known for its role in bone health, exhibits the potential to modulate cell proliferation and immune responses in skin carcinogenesis [142]. Antioxidants such as vitamin C and vitamin E are also pivotal, with their free radical scavenging properties aiding in skin cancer prevention [143,144]. While promising, the efficacy and safety of these vitamins, particularly at pharmacological levels, warrant further clinical investigation to optimize their use in NMSC therapeutic strategies (Figure 4).

### 5.1. Vitamin A

Vitamin A forms a wide group of substances, such as retinoids, retinol, retinyl palmitate, and beta-carotene, which can be derived from animal sources, such as eggs, milk, organ meats, cheese, or fish [141,145]. Of the substances mentioned above, retinoids are particularly potent in the treatment and prevention of non-melanoma skin cancer and have the ability to inhibit the development of skin carcinogenesis. They are vital for processes of cell turnover, immunity, proliferation, barrier functions, angiogenesis, and differentiation, playing a key role in the normal functioning and abnormalities of the skin. Moreover, through the thickening of the epidermis, they lower the amount of UV radiation that reaches the strata below [36,42]. However, retinoids contribute to more anti-cancer mechanisms. In terms of restraining cSCC, all-trans-retinoic acid (ATRA) represses the overexpressed in some aggressive cancers’ activator protein-1 and diminishes activation of STAT3 in keratinocytes, whereas STAT3 encourages keratinocyte proliferation, which potentially leads to its later malignancy. What is more, as retinoids play a role in angiogenesis, which is a vital case in cancer formation, retinoid treatment might efficiently inhibit skin cancer growth by impeding angiogenesis [36]. Moreover, via in vitro and in vivo testing to understand how retinol works, this mechanism of anti-cancer action presented by retinol is considered its primary [145]. Retinoids have shown effectiveness in treating certain types of skin cancers, such as BCC or cSCC. They have been proven beneficial in both prevention and therapy for certain types of skin cancers, with specific applications approved by the FDA and off-label uses [36].

### 5.2. Vitamin C

Vitamin C is an intracellular antioxidant that can be found in fruits and vegetables. In the available studies, it has been shown to provide protection against UV radiation and carcinogenesis. Moreover, scientists providing an investigation on cultured keratinocytes discovered that the combination of vitamins C and E had a beneficial effect. They observed that these vitamins were able to counteract the rise in ROS caused by UVB irradiation. Moreover, the combination of vitamins C and E provided protection against apoptosis triggered by UVB exposure. This suggests that the antioxidant properties of vitamins C and E may play a role in mitigating the harmful effects of UVB radiation on skin cells. However, it is important to note that further investigation is necessary to comprehend the mechanisms and practical implications of these findings fully. Vitamin C has also been found to have an impact on DNA repair. In a study involving human dermal fibroblasts treated with vitamin C, researchers observed an upregulation of genes related to DNA replication and repair. Additionally, the fibroblasts exhibited a faster repair process for DNA bases that had been damaged by oxidative stress. Vitamin C has been found to produce hydrogen peroxide in extracellular fluid, and this is believed to contribute to its anti-cancer properties through a process called the Fenton reaction. A recent study by Ngo et al. explored the pre-clinical evidence of how high doses of ascorbate can affect redox imbalance, epigenetic re-programming, and oxygen-sensing regulation, all potential mechanisms of its anti-cancer action. Interestingly, the metabolic effects of ascorbate have minimal impact on normal cells [146].

### 5.3. Vitamin E

Vitamin E, a lipid-soluble antioxidant, represents eight corresponding substances—four tocotrienols and four tocopherols. Its main sources are nuts, plant-based oils, soybeans, and wheat germ [146]. Vitamin E has been demonstrated to prevent the peroxidation of membrane lipids caused by ROS. In a study involving mouse keratinocytes, it was found that pre-treatment with vitamin E prior to UVB radiation resulted in a reduction in UVB-induced damage to the epidermis [146,147]. Similarly, in human fibroblasts exposed to UVA light, both vitamins C and E exhibited the potential to provide protection against the harmful effects of UVA radiation [146,148]. Included in the vitamin E group is tocopherol, which has been found to inhibit the activity of protein kinase C (PKC) and suppress tumor angiogenesis. However, the anti-cancer effect of vitamin E on NMSC has not yet been fully proven [149]. Several studies have indicated that there is no significant association between the serum level or intake of vitamin E and the risk of BCC [149]. Moreover, the randomized double-blinded trial of 7000 adults conducted by Argos et al. did not show any beneficial effect of treatment with vitamin E [150]. No studies have indicated any significant influence of vitamin E on NMSC.

### 5.4. Vitamin D

Vitamin D is a hormone that can be obtained from sources such as fish or mushrooms [151]. This compound possesses antioxidant as well as immunomodulatory properties, and its anti-cancer effect on NMSC should be confirmed [151]. What has been found in this field, according to in vitro animal models, is that vitamin D has the ability to modify cancer cell proliferation, cell death, differentiation, and tumor angiogenesis, making it a potential candidate agent for cancer regulation [151,152]. However, the question of whether vitamin D prevents cancer in humans or limits its progression is still unresolved and requires further investigation [142,151]. Various studies have considered vitamin D receptors to be tumor inhibitors in the skin due to their factors that are believed to alter cancerous cell functions [151]. However, studies have also suggested that higher serum levels of vitamin D3 are associated with a linear dose–response relationship. It is important to note that this association may be influenced by the dual effect of UVB radiation. While UVB exposure enables vitamin D synthesis, it can also lead to DNA damage, increasing the risk of skin cancer. Therefore, the relationship between vitamin D levels, UVB exposure, and skin cancer risk is complex and requires further investigation. What is more, another study indicated that suppressing strong carcinogens such as 7,12-dimethylbenzanthracene puts into effect a photoprotective function against UV radiation exposure, protecting against NMSC [42]. That is why vitamin D requires further research on its anti-cancer properties.

## 6. Alkaloids

**Figure 5:**
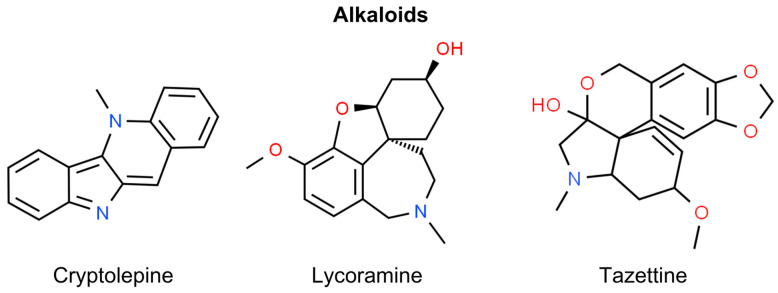
Chemical structures of selected alkaloids showing anti-NMSC effect.

Alkaloids are plant-derived substances with diverse structures primarily resulting from the biosynthesis of amino acids. These compounds can be found in small quantities in approximately 20% of plant species [153]. One of the most studied alkaloids in the context of skin cancer is cryptolepine, which is described below. However, it is worth noting the latest research by Castañeda et al., who investigated the chemotherapeutic and photoprotective potential of Amaryllidaceae Alkaloids from *C. jagus, Z. carinata, E. caucana*, and *H. elegans* in skin cancer. This study revealed the photoprotective potential of lycoramine and tazettine, which protects human keratinocytes from UVB-induced production of ROS and IL-6. It was also demonstrated that the alkaloid fraction of *Z. carinata* exhibited the highest potential as an anti-inflammatory agent (Figure 5) [154].

### Cryptolepine

Cryptolepine is a type of alkaloid obtained from the roots of a shrub, *Cryptolepis sanguinolenta*, which is found in Central and West Africa. It has shown numerous pharmacological and biological activities, amongst which anti-inflammatory activity can be highlighted. Through the inhibition of COX-2/PGE signaling, TNFα and iNOS cryptolepine have proven their anti-inflammatory activity in various animal model systems. Cryptolepine’s anti-tumor potential has been tested as the emergence and advancement of cancers are strongly linked to inflammation. The results have proven its cytotoxic potential against cancer, but the mechanism behind it has not been fully recognized. The research has been conducted to detect how criptolepine influences cancer cells using SCC-13 and A431 cell lines as an in vitro model. Treating NMSCC with cryptolepine significantly reduced topoisomerase activity, presumably due to DNA damage, which led NMSCC to decrease cell viability and their ability to form colonies and increase cell death through apoptosis. As topoisomerases are essential for DNA replication as well as cell proliferation, and their activity can be impaired because of cancer cells, it is important to inhibit topoisomerase functions and induce DNA damage, leading to apoptosis [155]. One of criptoleptine’s mechanisms of action that induces DNA impairment is the enhancement of the phosphorylation of ATM/ATR, BRCA1, Chk1/Chk2, and γH2AX. This compound also activated the p53 signaling pathway and downregulated cyclin-dependent kinases, cyclin D1, cyclin A, cyclin E, and proteins involved in cell division. What is more, the integrity of the mitochondrial membrane potential is compromised, and relief of cytochrome c occurs. All these actions end up with anti-proliferative and pro-apoptotic effects [93,155].

## 7. Terpenoids

**Figure 6:**
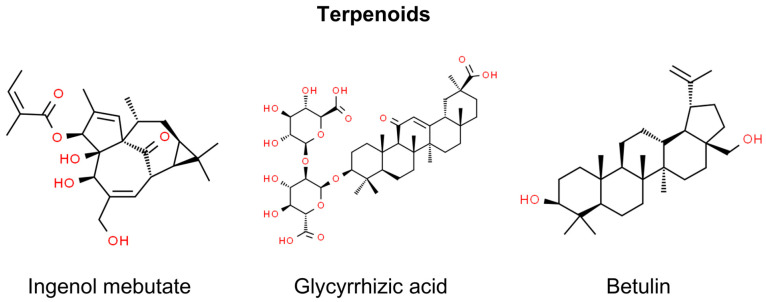
Chemical structures of selected terpenoids showing anti-NMSC effect.

Terpenoids, also known as terpenes or isoprenoids, are structurally assembled from five-carbon units, forming subclasses such as hemiterpenoids (C5), monoterpenoids (C10), sesquiterpenoids (C15), diterpenoids (C20), triterpenoids (C30), tetraterpenoids (C40), including carotenoids, and polyterpenoids (C5) [156]. They are found in various plants and organisms. Initially used in perfumes and flavorings, terpenoids are used nowadays in a wide range of applications, including medicine. The diterpene paclitaxel (Taxol), derived from *Taxus brevifolia*, is a notable commercial anti-neoplastic agent [156]. Certain terpenoids have been identified for their potential therapeutic roles in the realm of non-melanoma skin cancer prevention and treatment. These include ingenol mebutate, glycyrrhizic acid, betulin, betulinic acid, carnosic acid, carnosol, and geraniol. Notably, carnosic acid, carnosol, and geraniol have been researched for their effects on SCC of the head and neck rather than skin SCC (Figure 6) [157,158,159].

### 7.1. Ingenol Mebutate

The most investigated terpenoid in terms of non-melanoma skin cancer is inegnol mebutate, a diterpene ester isolated from the *Euphorbia peplus* plant [160]. Its anti-proliferative activity comprises mitochondrial swelling, disruption of membrane potential, and a decline in adenosine triphosphate synthesis that causes necrotic cell death [161]. Furthermore, residual dysplastic epidermal cells may be eliminated through neutrophil-mediated antibody-dependent cytotoxicity [162]. Notably, ingenol mebutate represents the natural compound that has progressed to clinical trials for the treatment of NMSC. In 2013, ingenol mebutate (Picato^®^) obtained marketing authorization from the European Medicines Agency (EMA) for the topical treatment of non-hyperkeratotic, non-hypertrophic AK in adults [163]. The EMA mandated a study to investigate the risk of SCC associated with ingenol mebutate. The results of the 3-year safety study revealed a higher incidence of skin cancer, leading the EMA to recommend suspending the license for ingenol mebutate gel in 2020 as a precaution pending further review. The Medicines and Healthcare products Regulatory Agency (MHRA) reported that the final study results among 484 patients indicated a higher occurrence of SCC in those using ingenol mebutate gel compared with imiquimod (3.3% vs. 0.4%) [164,165,166]. Stocks of the gel have been recalled in the UK and Canada, and clinicians have been advised to cease prescribing it and explore alternative treatments for AK [165,167].

### 7.2. Glycyrrhizic Acid

The terpenoid being researched in the field of skin cancers is glycyrrhizic acid (GlA). GlA is extracted from *Glycyrrhiza glabra* and demonstrates significant anti-tumor activity against melanoma by targeting the tumor microenvironment (TME) and modulating immune responses. GlA’s mechanisms include inducing apoptosis in melanoma cells and reducing their proliferation [168]. This is achieved by upregulating pro-apoptotic factors such as Bax and caspase 3 and downregulating the anti-apoptotic factor Bcl2. Altering cytokine profiles within the TME, GlA shifts the balance from anti-inflammatory to pro-inflammatory cytokines, promoting a Th1 immune response conducive to tumor rejection. GlA impacts T Regulatory Cells (Tregs) and Myeloid-Derived Suppressor Cells (MDSCs) by inhibiting STAT3 phosphorylation, a crucial factor in their immunosuppressive function. This leads to reduced Treg markers (Foxp3, GITR, and CTLA4) and the downregulation of MDSC-associated factors (Cox2, PGE2, and Arginase 1) [168]. When combined with *Mycobacterium indicus pranii* (Mw), an immunomodulator, GlA shows enhanced effectiveness, particularly against advanced-stage melanoma. [168] GlA’s multifaceted approach against melanoma positions it as a potential candidate for melanoma immunotherapy and cancer treatment.

GlA provides protective effects to skin cells against the stresses caused by UVB radiation, which is responsible for most non-melanoma skin cancers. Through various pathways, GlA demonstrates its photoprotective effects against UVB radiation-induced damage in primary human dermal fibroblasts (HDFs). GlA significantly reduces cell death in UVB-irradiated HDFs and prevents UVB-induced Cyclobutane Pyrimidine dimers (CPDs) formation, a major form of DNA damage caused by UVB [169]. It also diminishes DNA fragmentation, suggesting its role in protecting HDFs from UVB-induced DNA damage. GlA exhibits strong antioxidant properties by quenching ROS generated due to UVB exposure [169]. GlA relieves HDFs from the oxidative stress-mediated endoplasmic reticulum (ER) stress [169]. It also influences the process of autophagy—a cellular degradation and recycling process. Moreover, GlA treatment leads to changes in the expression levels of autophagy-related proteins, such as p62, BECN1, mTOR, and ATG7, especially in the initial hours following UVB irradiation [169]. GlA downregulates key DNA damage marker proteins, such as ATM, ATR, Chk1, Chk2, DDB2, P53, and χH2AX, in HDFs irradiated with UVB. Additionally, GlA stabilizes the AKT/PTEN axis, which is known to be affected by UVB irradiation. GlA stabilizes this axis, indicating its role in protecting cells against UVB-induced effects [169]. The use of chloroquine, an autophagy inhibitor, potentiates UVB-induced DNA damage in GlA-treated HDFs, while rapamycin, an autophagy activator, enhances GlA’s protective effect against UVB-induced damage [169].

### 7.3. Betulin and Betulinic Acid

Betulin and betulinic acid, naturally occurring triterpenes commonly derived from the bark of birch trees, are known for their protective properties against conditions such as oxidative stress, inflammation, and cancer [93,170]. Betulin is classified as a lupine-type compound and is distinguished by the presence of an isopropylidene group and a five-membered ring [171]. These compounds promote the differentiation of normal human keratinocytes and induce cytotoxic, anti-proliferative, and apoptotic responses in tumor cells through the direct interaction with mitochondria by rendering the mitochondrial membrane permeable, leading to the release of apoptogenic proteins into the intermembrane space. Interestingly, this apoptotic process is triggered independently of the p53 gene and excludes the CD95 receptor. Additionally, it modulates the action of the NFkB transcription factor and activates the Nrf2 pathway [172,173,174].

In the context of NMSC, the anti-cancer properties of betulinic acid, betulin, and its new esters containing lysine or ornithine side chains were investigated in an in vitro study. It was indicated that newly modified compounds originating from betulin hold significant therapeutic potential for actinic keratosis, and the most significant benefit is that they are safe for normal human keratinocytes [175]. Betulin has also been confirmed as a potent anti-mutagenic agent against skin carcinogenesis in in vivo research. In experiments using chemically damaged mice skin, a topical formulation with betulin nanoemulsion was tested. The skin damage could lead to significant pathologies, including neoplasms. Betulin reduced skin lesions and irritation, notably decreasing erythema, and exhibited inhibitory effects on the initiation and promotion of skin tumors. Additionally, betulin increased the respiratory function of isolated liver mitochondria in a two-stage model of skin carcinoma in mice. It also showed the potential to reduce damage in vital organs, such as the liver, due to the slow penetration of carcinogens applied to the skin surface, resulting in toxic effects by carcinogen penetration [176].

## 8. Isothiocyanates

**Figure 7:**
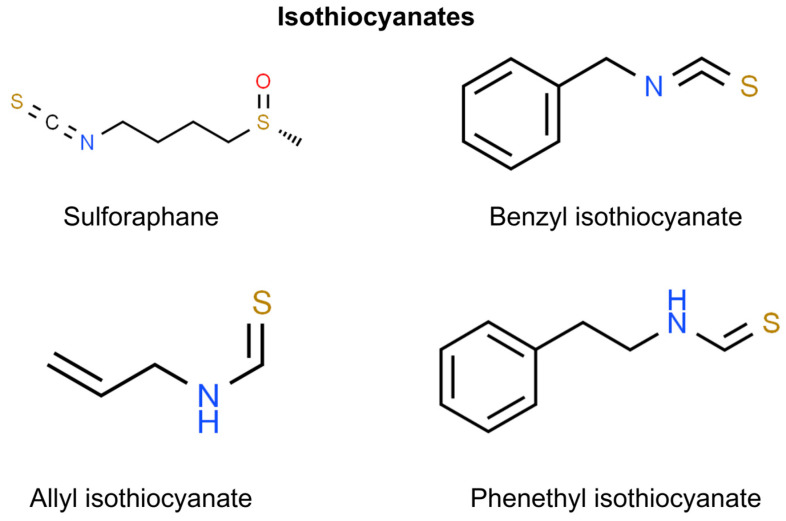
Chemical structures of selected isothiocyanates showing anti-NMSC effect.

Isothiocyanates are compounds derived from glucosinolates found in cruciferous vegetables, such as broccoli, cauliflower, and kale. When these vegetables are chopped, chewed, or otherwise processed, an enzyme—myrosinase—transforms glucosinolates into isothiocyanates [177]. These compounds are known for their potential health benefits, including anti-cancer properties. They may work by altering the mechanisms of carcinogenesis, such as by inducing apoptosis in cancer cells, inhibiting angiogenesis, and protecting cells from DNA damage [178]. In the context of non-melanoma skin cancers, compounds such as sulforaphane, benzyl isothiocyanate, allyl isothiocyanate, and phenethyl isothiocyanate are recognized for their potential preventive or therapeutic properties. It is essential to acknowledge, however, that investigations into benzyl isothiocyanate, allyl isothiocyanate, and phenethyl isothiocyanate mainly concentrate on their effects on head and neck or esophageal SCC and not on skin SCC (Figure 7) [179,180,181].

### Sulforaphane

Sulforaphane (SFN), a constituent of the isothiocyanate family, is notably present in significant amounts in both broccoli and its sprouts. This family encompasses sulfur-containing compounds, including allyl, benzyl, phenylethyl, isopropyl, and methyl thiocyanate. These compounds are widely distributed among cruciferous vegetables, such as cauliflower, kale, watercress, and cabbage [182,183]. Research indicates that SFN demonstrates efficacy in the prevention or reversal of various neoplasms, including breast, colon, prostate, and lung cancer and melanoma [184,185,186,187].

SFN exhibits differential effects on both normal and cancer cells. In the latter, SFN induces cell cycle regulatory proteins such as p21 and p16, leading to apoptosis and inhibition of cell proliferation [188]. This upregulation is also attributed to the inhibition of histone deacetylase (HDAC) expression and its activity in cancer cells. The combination of green tea polyphenols (GTP), a dietary DNMT inhibitor, with SFN was shown to reactivate p21 and Klotho genes through histone acetylation [189]. On the contrary, normal keratinocytes show resistance to the SFN-induced inhibition of HDAC and cell proliferation compared with cancer cells [21].

SFN exhibits potent anti-cancer effects, including against skin cancer, by inhibiting HDACs and DNA methylation [190]. SFN reactivates Nrf2, a transcription factor for antioxidant enzymes, by down-regulating DNA methyltransferases (DNMTs) and HDACs in JB6 mouse skin epidermal cells exposed to TPA, thus suppressing TPA-induced malignant transformation [191]. Through the involvement of the Nrf2-dependent mechanism, the topical application of SFN in mouse skin results in increased glutathione (GSH) and glutathione S-transferase 4 (GST4) syntheses, which inhibit skin mutagenesis [192].

Another anti-tumor mechanism of SFN relating to epigenetic regulators includes polycomb group proteins (PcG), which are implicated in chromatin remodeling and the inhibition of gene expression. Elevated expression of PcG proteins, such as Bmi-1, Ezh2, and SUZ12, is observed in various skin cancers [190]. High Bmi-1 expression promotes transcriptional inactivation of regulatory cell cycle proteins, such as ARF and INK4A, by recruiting histone methylation complexes (PRC2 and PRC1). Treatment with SFN inhibits cell proliferation by reducing the expression of Bmi-1 and Ezh2.

**Figure 8:**
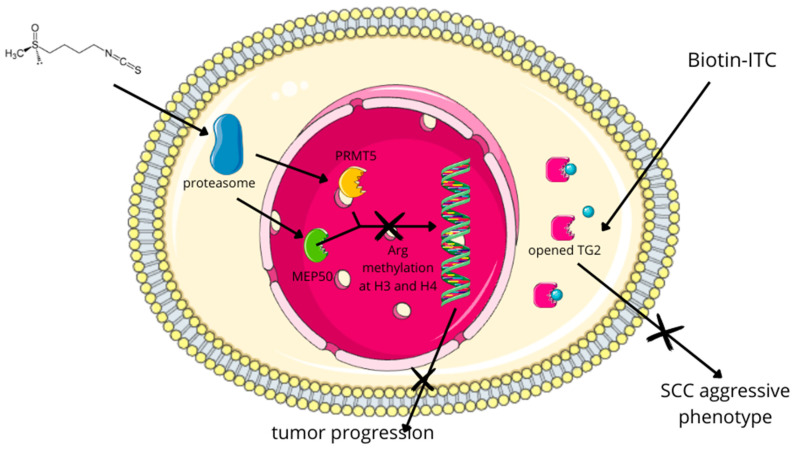
In SCC, exposure to SFN hinders cancer progression and in vivo metastasis by diminishing arginine methylation at histone 3 (H3). This reduction involves SFN-induced proteasomal degradation of arginine N-methyltransferase 5 (PRMT5) and methylosome protein 50 (MEP50). Both enzymes are responsible for arginine methylation at H3 and H4, respectively, leading to decreased levels of dimethylated arginine 3 at H4 (H4R3me2) [193]. Moreover, using a biotin-tagged SFN analog (Biotin-ITC) and kinetic analysis, it was showed that SFN covalently binds to recombinant type 2 transglutaminase (TG2), irreversibly inhibiting its transamidase activity. This induces an open/extended conformation and partially inhibits GTP binding, which is crucial for maintaining the aggressive SCC phenotype [194].

Among non-melanoma skin cancers, SCC has been the most frequently investigated in the context of SFN treatment. In SCC, exposure to SFN hinders cancer progression and in vivo metastasis by diminishing arginine methylation at histone 3 (H3). This reduction involves SFN-induced proteasomal degradation of arginine N-methyltransferase 5 (PRMT5) and methylosome protein 50 (MEP50). Both enzymes are responsible for arginine methylation at H3 and H4, respectively, leading to decreased levels of dimethylated arginine 3 at H4 (H4R3me2) [193]. Moreover, using a biotin-tagged SFN analog (Biotin-ITC) and kinetic analysis, it was shown that SFN covalently binds to recombinant type 2 transglutaminase (TG2), irreversibly inhibiting its transamidase activity. This induces an open/extended conformation and partially inhibits GTP binding, which is crucial for maintaining the aggressive SCC phenotype (Figure 8) [194]. Furthermore, the combined therapy of SFN and cisplatin for SCC suppressed tumor formation and reduced the population of cancer stem cells within the tumor [195].

## 9. Cannabinoids

**Figure 9:**
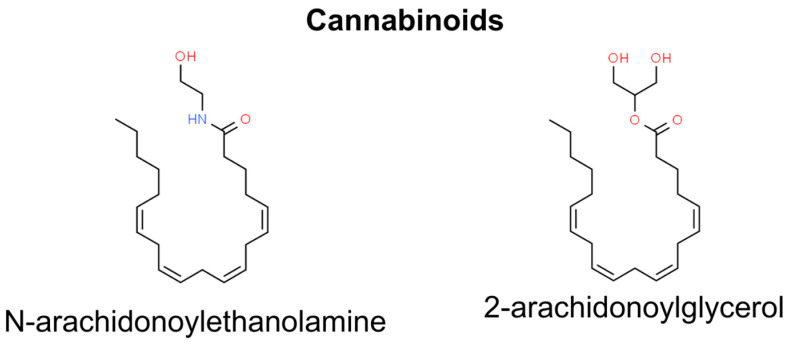
Chemical structures of selected cannabinoids showing anti-NMSC effect.

Both naturally occurring and synthetic cannabinoids have demonstrated their ability to influence a wide range of biological actions in various cancer types [196]. Moreover, recent research highlighted the important role of the endocannabinoid system in regulating skin functions and proposed cannabinoids as potential therapy and prevention for skin cancer [197]. The endocannabinoid system consists of two cannabinoid receptors: cannabinoid receptor 1 (CB1) and 2 (CB2). Both CB1 and CB2 receptors are G-protein coupled receptors, and they interact with endogenous ligands, namely N-arachidonylethanolamine (AEA) for CB1 and 2-arachidonylglycerol (2AG) for CB2, both derived from arachidonate [198]. Findings have indicated that cannabinoid receptor activation by chemical carcinogens and UVB radiation can boost tumor growth, whereas externally administered cannabinoid compounds trigger tumor cell death. These contrasting effects may be connected with the concentration-dependent impact of cannabinoids on skin cancer cells. Endogenous endocannabinoids at nanomolar levels linked to carcinogen exposure promote tumor development, while exogenous cannabinoids at micromolar levels reduce it [199,200]. In addition, recent studies showed that the endocannabinoid Arachidonoyl-ethanolamine (AEA) causes apoptosis in multiple tumor types, especially with malignancies that overexpress cyclooxygenase-2 (COX-2). It has been demonstrated that COX-2 metabolized AEA to J-series prostaglandins, and that consequently causes endoplasmic reticulum stress. NMSCs and various epithelial tumors exhibit elevated levels of COX-2, distinguishing them from normal cells. Therefore, AEA could be a potential topical agent for the elimination of these types of skin cancer (Figure 9) [201,202].

## 10. Carotenoids

**Figure 10:**
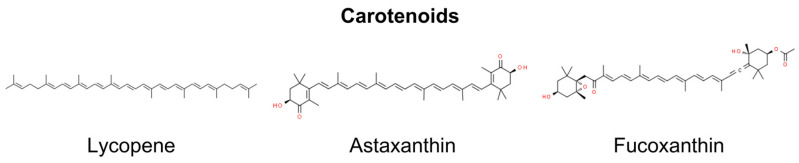
Chemical structures of selected carotenoids showing anti-NMSC effect.

Carotenoids are bioactive antioxidant compounds widely present in plants. Carotenoids can be categorized based on their polarity. There are two distinguished groups: carotenes (such as β-carotene and lycopene) and xanthophylls (such as lutein, fucoxanthin, and astaxanthin), which are more polar due to the inclusion of hydroxy- or keto-functional groups in their molecular structures [203,204]. As mentioned above, one of the most significant risk factors for the development of skin cancers is exposure to UVR. Many of the detrimental effects induced by it are primarily mediated through oxidative stress, which disrupts signal transduction pathways [1]. Given their photoprotective and antioxidant properties, carotenoids should be considered as preventive factors in the development of skin tumors. Their mechanisms include activating the antioxidant response element transcription system and enhancing gap junctional cell–cell communication by regulating connexin gene expression [203]. Research indicates that antioxidant carotenoids such as lycopene, fucoxanthin, and astaxanthin may play a significant role in skin photoprotection (Figure 10).

### 10.1. Lycopene

Lycopene, an antioxidant derived from plants such as tomatoes, watermelons, red carrots, and papayas, exhibits potential bioactivity and notable health benefits. This compound comprises eight isoprene units linked together, forming a tetraterpene structure. Additionally, it contains a total of 11 linear double bonds. Research has focused on its anti-cancer properties, including inhibiting apoptosis and cell proliferation and affecting various signal transduction pathways. Several studies highlighted its biological activity concerning skin cancers and photoprotection [205,206]. It has been shown that regular consumption of lycopene can protect the human skin from the effects of UVR, including erythema, alterations in the extracellular matrix, and damage to mitochondrial DNA [207].

In chemically induced cutaneous tumor mice and cell models, lycopene reduced both the incidence and multiplicity of cutaneous tumors and inhibited the tumorigenesis of normal cutaneous cells during the promotion phase. This effect was associated with activating antioxidant enzymes and the transcription factor Nrf2 (nuclear factor erythroid 2-related factor 2). Additionally, lycopene increased the expression of the autophagy protein p62, leading to the degradation of Keap1 (Kelch ECH associating protein 1), the main protein responsible for keeping Nrf2 in the cytoplasm [208].

### 10.2. Astaxanthin and Fucoxanthin

Astaxanthin (ASX) and fucoxanthin (FX) are unique marine carotenoids known for their remarkable antioxidant qualities. Astaxanthin is composed of a polyene chain connecting two terminal β-ionone-type rings. The molecule features two asymmetric carbons situated at the 3,3′-position of the β-ionone ring, and each end of the molecule is equipped with a hydroxyl group. Additionally, oxygen is incorporated into the ring system in the form of hydroxyl and keto groups [209]. Fucoxanthin possesses a distinctive structure, featuring an uncommon allenic bond, nine conjugated double bonds, a 5,6-monoepoxide, and several oxygenic functional groups, such as hydroxyl, epoxy, carbonyl, and carboxyl moieties [210]. These carotenoids have demonstrated exceptional effectiveness in mitigating various types of photodamage. This is attributed to its antioxidant capabilities and the initiation of alternative pathways. [211,212]. Research suggests the potential health-promoting effects of ASX and FX in the prevention of skin cancer.

It has been indicated that FX and AX play a role in activating the Nrf2 signaling pathway, prompting the epigenetic demethylation of CpG sites within Nrf2 and demonstrating the ability to inhibit the TPA-induced transformation of mouse skin JB6 P+ cells [213]. Moreover, it has also been shown that in human keratinocytes, FX treatment augments cellular antioxidant defense by inducing Nrf2-driven expression of enzymes involved in GSH synthesis via PI3K/Akt signaling [214].

It has been shown that prior exposure to 5 µM of ASX in human keratinocytes (HaCaT) 24 h before UVB exposure or the topical application of a 0.02% ASX gel following chronic UVB irradiation in male Wistar mice (administered three times per week for 4 weeks), demonstrated the ability to hinder oxidative DNA damage [215,216]. Rao et al. investigated astaxanthin mono- (AXME) and diesters (AXDE) for anti-cancer potency in rat cancer models. AXDE and AXME exhibited a significant reduction in UV-7,12-dimethylbenz(a)anthracene (DMBA)-induced tumor incidences, reaching 96% and 88%, respectively. This effectiveness was notably higher compared with the reduction achieved with ASX alone, which was at 66%. It has been suggested that this is due to the better bioavailability of ester compounds [217].

## 11. Ceramides

**Figure 11:**
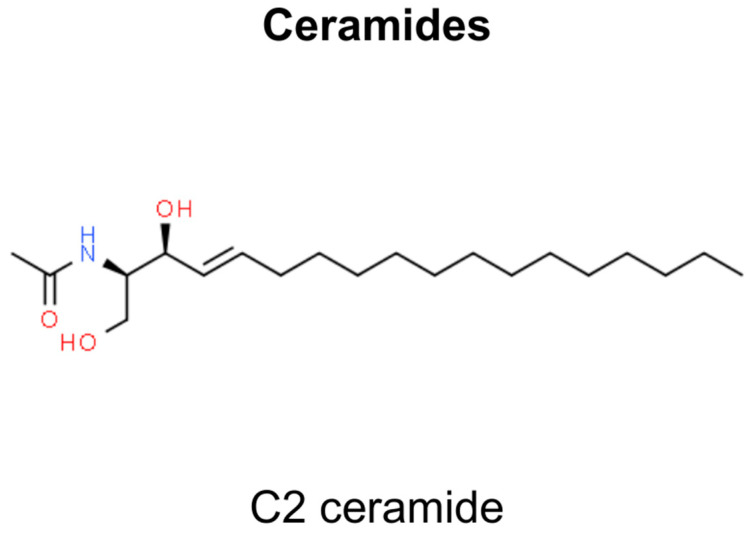
Chemical structures of C2 ceramide showing anti-NMSC effect.

Ceramides, the primary constituents among sphingolipids, are synthesized within cells from sphingomyelin breakdown or via the de novo synthesis pathway involving serine and palmitoyl-CoA. These compounds are integral lipid molecules in cell membranes, which are crucial for maintaining the skin’s barrier and facilitating cellular processes such as apoptosis, growth, and differentiation [218]. Additionally, they play a role in diseases such as cancer and neurodegenerative disorders [219,220]. While present in some foods and skincare products, these external ceramides differ from those synthesized in cells. Synthetic variants such as C2 ceramide are created to emulate natural ceramides’ structure and functions, which is important for research and therapeutic applications, especially in regulating cell cycle, survival, proliferation, differentiation, and immune responses, often through the modulation of proteins like Protein Kinase C (PKC) isoforms, including atypical Protein Kinase C zeta (PKCζ) (Figure 11) [221,222].

C2 ceramide has a therapeutic impact, especially on melanoma cells. The generic structure of C2 ceramide, also known as N-acetylsphingosine, comprises two primary components—sphingosine backbone and fatty acid chain, which in C2 ceramide is an acetyl group containing two carbon atoms. These two components are linked together by an amide bond. The anti-melanoma efficacy of C2 ceramide focuses on its impact on the tumor microenvironment (TME). C2 ceramide induces apoptosis in melanoma cells by upregulating PKCζ, pro-inflammatory cytokines, and signaling factors, reducing melanoma cell viability and proliferation [222]. A key aspect of its action is the inhibition of Akt phosphorylation, as PKCζ, upregulated by C2 ceramide, plays a crucial role in this process. Akt, a protein integral to cell survival pathways, when inhibited, results in increased apoptosis of melanoma cells [222]. C2 ceramide facilitates apoptosis and cell cycle inhibition in melanoma cells via PKCζ-mediated endogenous ceramide production. Concurrently, it modulates macrophages within the tumor microenvironment, orchestrating a phenotypic transition from M2 to M1, a process governed by PKCζ characterized by altered cytokine secretion and enhanced antigen-presenting capacity [222]. C2 ceramide treatment attenuates angiogenic factors, including VEGF, VEGFR1, VEGFR2, and HIF1α, normalizing the TME and promoting Th1-type responses, thereby augmenting cytotoxic T cell activity against melanoma. It also mediates direct Akt-PKCζ interactions, elucidating intricate regulatory pathways [222].

SCC appears to be influenced by C2 ceramide as well. The molecular pathways of ceramide in inducing apoptosis in SCC cells involve several interconnected processes. C2 ceramide effectively induces apoptosis in human SCC cells (HSC-I) [223]. This intrinsic apoptotic effect was confirmed through various methods showing dose-dependent toxicity and morphological changes typical of apoptosis, including chromatin condensation, internucleosomal DNA fragmentation, and nuclear fragmentation. The specificity of ceramide’s action is also highlighted by comparing the effects of C2 ceramide with C2 dihydroceramide and noting that only C2 ceramide induced apoptosis [223]. This specificity underscores the critical role of the double bond in ceramide for its apoptotic activity. Additionally, a role for ceramide in terminal differentiation was proposed. It is hypothesized that the increase in intracellular ceramide associated with epidermal differentiation might be involved in the terminal differentiation of keratinocytes, akin to a specialized form of apoptosis. This leads to potential therapeutic implications, suggesting that ceramide or its analogs might have applications in treating hyperproliferative skin disorders, including skin cancers.

The apoptotic role of ceramide in both melanoma and non-melanoma cells highlights its potential to regulate cell death and differentiation, particularly in skin pathologies. It opens avenues for considering ceramide and its analogs in therapeutic strategies for skin-related disorders and cancers (Table 2).

## 12. Wound Healing and Skin Regeneration

To date, radical tumor excision remains the most efficient approach for the treatment of skin cancer. However, surgical interventions can be significantly damaging to the skin. Therefore, post-surgery management should include the use of wound dressings to promote skin regeneration and prevent tumor recurrence. Ideal candidates for regenerative applications in skin cancer could be wound dressings consisting of biopolymers for regeneration and natural anti-cancer agents to prevent tumor recurrence.

Betulin is also recognized for its potential to promote wound healing. The process of wound healing is a complex combination of cellular and molecular events associated with tissue regeneration and inflammation in the initial phase. Studies have confirmed that betulin accelerates the initiation of the inflammatory phase by temporarily increasing the expression of pro-inflammatory mediators. Importantly, this transient overexpression does not result in prolonged inflammation. Furthermore, betulin facilitates the migration of keratinocytes, a crucial aspect of the second phase of wound healing [224,225].

In vitro and in vivo studies have affirmed its potential to promote tissue regeneration by augmenting granulation formation, thereby facilitating wound healing. Additionally, it demonstrates bacteriostatic effects [226]. Lakshmanan et al. established the therapeutic potential of resveratrol-loaded polymeric fibrous scaffolds in the initial stages of the wound healing process using a mouse model of ischemic wounds. This study demonstrated that the expression of Bcl2 in the healing wound edges post-treatment with the resveratrol-loaded scaffold confirmed the anti-apoptotic effect mediated by this compound [227]. In a clinical study, the topical application of an antioxidant blend comprising resveratrol, baicalin, and vitamin E on photodamaged skin resulted in a noteworthy enhancement of skin elasticity, firmness, laxity, and reduction in hyperpigmentation [228].

Apart from the putative anti-cancer effect of flavonoids through their UV-blocking activity, flavonoids may be beneficial in accelerating wound healing processes via the regulation of numerous pathways, notably Wnt/β-catenin, Hippo, TGF-β, Hedgehog, c-Jun, Nrf2/ARE, NF-κB, MAPK/ERK, Ras/Raf/MEK/ERK, PI3K/Akt, and NO [229].

Vitamin A deficiency has long been associated with delayed wound healing, leading to impaired wound closure, decreased collagen synthesis, and cross-linking. Vitamin A stimulates epidermal turnover, accelerates re-epithelialization in wounded skin, and restores epithelial structure. The effectiveness of retinoids, whether applied topically or ingested orally, is comparable. Retinoids act by binding to specific receptors in the cytoplasm and nucleus, influencing cell division, differentiation, RNA and protein synthesis, and lysosome-membrane stabilization [230].

Vitamin C significantly influences tissue repair and regeneration, particularly in collagen synthesis, providing tensile strength to newly formed collagen. Its antioxidant properties are essential for removing and neutralizing oxidants in the body, particularly in the epidermis. Vitamin C has been linked to an increased proliferation of dermal fibroblasts, a critical function for wound healing. Given the increased turnover at wound sites and the potential for inflammation to deplete vitamin C, supplementation may be beneficial to the healing process. Vitamin C deficiency is associated with delayed healing and impaired subcutaneous healing [143].

The properties of vitamin E in relation to wound healing are manifested through its multifaceted roles as an antioxidant, its impact on connective tissue growth factor (CTGF), its modulation of methicillin-resistant Staphylococcus aureus (MRSA), and its influence on gene transcription [231].

Curcumin has historically been used in Ayurveda for its healing properties, as it exhibits anti-inflammatory effects and influences various stages of healing. While curcumin has demonstrated effectiveness in eradicating ROS and promoting collagen deposition, granulation tissue formation, and wound contraction, its limited solubility, rapid metabolism, and short plasma half-life hinder its application in wound healing.

Nanotechnology has emerged as a promising approach for overcoming these limitations and enhancing wound healing by facilitating the targeted delivery of curcumin at wound sites. This review underscores the potential of curcumin and its nanoformulations, including liposomes, nanoparticles, and nanoemulsions, in wound healing [232].

Terpenoids are also recognized for their role in promoting wound healing, which is primarily attributed to their astringent and anti-microbial properties. These properties contribute to wound contraction and an enhanced rate of epithelialization [233].

Hemati et al. demonstrated that nanoliposomes containing sulforaphane as a nano-drug delivery system encapsulated within the scaffold hydrogel system improve wound healing [234].

## 13. Reactive Oxygen Species and NMSC

Excessive generation of ROS and a deficiency in antioxidant defense mechanisms, known as oxidative stress, have been associated with the onset of NMSC. Some of the mentioned compounds, such as flavonoids, vitamins, certain alkaloids, curcumin, carotenoids, sulforaphane, and betulin, possess photoprotective and antioxidative properties, effectively reducing the concentration of free radicals in various ways. One key role involves their impact on the Nrf2 pathway, whose activation induces the action of antioxidant enzymes. During the early stage of UV-induced skin carcinogenesis, Nrf2 activation promotes the proliferation of normal cells, outnumbering precancerous cells and preventing their expansion and mutation. The antioxidant defense mediated by Nrf2, which is capable of handling excessive ROS formation in the human body, may be valuable in preventing ROS-mediated neoplastic transformation in the skin [205,206].

## 14. Methodology

In this narrative review, our focus was on obtaining a comprehensive understanding of the role of natural compounds in the prevention and treatment of NMSC. Our overarching hypothesis posited that specific natural agents hold potential against NMSC. To systematically gather the relevant scientific literature, the authors thoroughly searched multiple bibliographic databases, including PubMed, Google Scholar, and Web of Science. This search adhered to a predefined hypothesis and inclusion and exclusion criteria, considering only the most relevant and current research findings for inclusion in this review.

The primary search strategy involved using specific keywords, such as “natural compound*” OR “natural agent*” OR “natural substance*” AND “non-melanoma skin cancer*” OR “NMSC” OR “squamous cell carcinoma” OR “SCC” OR “basal cell carcinoma” OR “BCC” OR “actinic keratosis” OR “AK”. Inclusion criteria were limited to articles published in English between 2014 and 2023, including clinical studies, in vivo and in vitro investigations, systematic reviews, and meta-analyses. The final selection of studies for review was achieved through a consensus-driven decision-making process by two authors, resulting in the inclusion of 195 resources. Articles were excluded if they did not align with the specified criteria or redundantly reiterated combinations of phrases.

## 15. Conclusions

This review highlights the potential role of naturally derived agents in future NMSC prevention and treatments, summarizing the most recent studies on various compounds of natural origin with potential anti-cancer properties. We aimed to investigate the potential anti-tumor properties of natural compounds, specifically in the context of NMSC, contrasting with the extensive body of evidence already available regarding the use of natural agents in addressing melanoma. Additionally, we sought to highlight the growing imperative for novel preventive and treatment strategies for NMSC, given its elevated incidence and mortality rates globally.

Although the economic aspect of using natural compounds is traditionally acknowledged, the transition to commercialization introduces concerns regarding resource depletion, consistency in quality, and safety issues. The incorporation of semi-synthetic methods in medical compound production exacerbates cost considerations.

The phenomenon of cancer patients engaging in self-medication with natural products, often lacking scientific substantiation, sheds light on the necessity to bridge the divide between traditional and conventional medical approaches. Challenges in assessing the quality of complementary and alternative medicines stem from safety apprehensions, absence of regulatory oversight, and formulation variations.

We advocate for clinicians to engage in pharmaco-vigilance practices, initiate large, randomized trials, actively inquire about natural compounds’ use among patients, systematically assess potential side effects, and counsel accordingly. Notwithstanding emerging animal studies, there remains a dearth of evidence from human clinical trials supporting the efficacy of any natural compound in the treatment of skin cancer, NMSC especially. Future research endeavors are essential to elucidate the precise role of natural agents in this domain.

## Figures and Tables

**Table 1:** Summary of basic NMSC characteristics.

	Basal Cell Carcinoma (BCC)	Squamous Cell Carcinoma (SCC)	Actinic Keratosis (AK)
Appearance	Various clinical types, with the nodular type as the most frequently observed variant, usually shiny, pearly papule with a smooth surface, rolled borders, telangiectatic surface vessels [6,30]	Firm, smooth, or hyperkeratotic papule or plaque, possible central ulceration [30]	Macules, papules, or hyperkeratotic plaques with an erythematous background [31]
Metastatic potential	Less than 0.1% [5]	From 0.3% to 3.7% [5]	Pre-malignant lesion [32]
Site of development	Sun-exposed areas of the body, including the scalp, face, and forehead [6]	Sun-exposed areas of the body, de novo or from a predisposing lesion, including AK [11]	Sun-exposed areas of the body, including the face, neck, dorsum of the hands, forearms, and lower legs [32]
Survival rate	About 100% [33]	95% [33]	About 100% [34]
Mutation statues	Hh pathway genes, particularly PTCH and SMO [8]	TP53, p16INK4a genes, PTEN [12,13,18]	TP53, p16INK4a genes, MYC, EGFR genes [26,27,28]

**Table 2:** Summary of preventive or therapeutic potential of natural compounds regarding NMSC. In the column labeled “Origin”, the natural source of the generic substances used in the research is specified.

Compound	Origin	Study	Conditions	Prevention /Treatment
Curcumin	Curcuma longa	Curcumin treatment in HaCaT cells significantly attenuated acute UVB-induced damage by reducing lactate dehydrogenase release, intracellular ROS, and DNA damage while upregulating phase II detoxifying enzymes and promoting DNA repair activity. Topical curcumin application inhibited UVB-induced inflammation, collagen disruption, and lipid peroxidation while promoting Nrf2 nuclear accumulation in hairless mice skin [81].	In vitro (HaCaT cells) and in vivo (uncovered hairless mice)	Prevention
Curcumin-loaded nanopatterned films	Curcuma longa	The curcumin-loaded nanopatterned films presented good cytotoxicity against the SCC cell model [88].	In vitro (A431 cell line)	Treatment
Curcuminoid complex	Curcuma longa	Pre-treatment application of the curcuminoid complex hindered UVB-induced FGF-2 expression, FGF-2-mediated cell proliferation, progression, and colony formation, along with suppressing mTORC1 and mTORC2 activation, as well as FGFR2 phosphorylation, in JB6 epithelial cells. Oral application of the curcuminoid complex on mice inhibited UVB-induced epidermal hyperplasia and hyperproliferation [83].	In vitro (murine epidermal JB6 P+ cells) and in vivo (SKH-1 mice)	Prevention
Curcumin-loaded liposome-siRNA	Curcuma longa	The topical application of a curcumin-loaded liposome-siRNA complex induced a more pronounced inhibition of SCC cell model growth and apoptosis events compared with the control group [87].	In vitro (A431 cells)	Treatment
Gallic acid	Gallnuts, sumac, witch hazel, tea leaves, oak bark	In BCC, gallic acid reduces cell migration and proliferation. In SCC, gallic acid reduces cell migration and proliferation, promotes cell death and significantly lowers HSP90AB1 levels in cancer cells [92].	In vitro (frozen samples of BCC, SCC from the Human Biological Bank)	Treatment
Silymarin	Silybum marianum	Silymarin induces an anti-inflammatory response, protects against thymine dimer formation induced by UVB radiation, triggers apoptosis in damaged cells, encourages the repairment of DNA, and targets aberrant signaling pathways [96].	In vitro (various types of cell lines, including A431 and HaCaT cells)	prevention
Resveratrol + Ursolic Acid	Plant Resveratrol: grapes Ursolic Acid: Arctostaphylos uva ursi	The administration of ursolic acid + resveratrol during skin tumor promotion caused by TPA showed greater tumor development inhibition than using each agent alone [109].	In vivo (Hsd: ICR (CD-1) mice)	Prevention
Quercetin	Onions, grapes, berries, cherries, broccoli, and citrus fruits	Quercetin reduced UVB-induced skin edema and blocked the NF-Κβ/COX-2 signaling pathway [116].	In vitro (HaCaT cells)	Prevention
Kaempferol	Broccoli, cabbage, kale, beans, endives, leek, tomatoes, strawberries, and grapes	Kaempferol delayed tumor growth by targeting RSK2 and MSK1 [119].	Animal model (SKH-1 hairless mice)	Prevention
Epigallocatechin gallate (EGCG)	Green tea	EGCG exhibits anti-proliferation potential by inactivating β-catenin signaling and reduces targets of β-catenin signaling, including MMPs, c-Myc, and VEGF. It decreases the levels of COX-2 and PGE2 as well [121].	In vitro (A431 and SCC13 cells)	Prevention
Gallocatechin gallate (GCG)	Green tea (epimer of EGCG)	GCG protects skin from UVB-induced photodamage, improves skin elasticity, and increases the number of collagen fibers, as well as inhibits aberrations in mitochondria and the formation of melanosomes [123].	Animal model (BALB/c hairless mice)	Prevention
Apigenin	Parsley, chamomile, celery, vine spinach, artichokes, and oregano	Apigenin suppresses the expression of IKKα epithelial-mesenchymal transition (EMT) was diminished, indicating potential anti-metastatic activity [127].	In vitro (PDVC57 and PB cells)	Prevention
		Apigenin may induce apoptosis by downregulating sulfiredoxin expression and activating the MAPK signaling pathway 2022 [125].	In vitro (JB6 cells)	Prevention
Daidzein	Soybeans	Secondary daidzein metabolite reduced the overexpression of COX-2 and MMP-9 by downregulating MAPK pathway signaling in particulate matter-exposed HaCaT keratinocytes [135].	In vitro (HaCaT cells)	Prevention
Genistein	Soybeans	Genistein suppresses UVB-induced inflammatory cytokines CXCL1, IL-1, MIF, and PLANH1 in vivo. Topical-administrated genistein decreased the number of skin folds and wrinkles induced by UVB in animal models. Diet reach in genistein in human participants significantly reduced the severity of UVB-induced wrinkling [136].	In vitro (HaCaT cells), in vivo (Sprague–Dawley rats, humans)	Prevention
		Genistein has an anti-nitrosative effect, preventing UVB-induced cell damage [137].	In vivo (hairless HRS/J mice)	Prevention
Biochanin A	Red clover, chickpeas, soybeans	Biochanin A inhibited the expression of UV-induced COX-2 [139].	In vitro (HaCaT cells, JB6 P+ mouse skin)	Prevention
Vitamin A	Eggs, dairy products, meat organs, fish	Vitamin A represses overexpressed activator protein-1, inhibits tumor angiogenesis, and diminishes activation of STAT3 [36].	In vitro (human BCC cell line—BCC-1/KMC) and in vivo (Ptch1+/− mice; humans)	Prevention, treatment
Vitamin C	Brussels sprouts, bell peppers, and berries, such as strawberries and blackcurrants	Vitamin C has antioxidant properties, influences DNA repair and replication, and produces hydrogen peroxide in extracellular fluid [146].	In vitro (Colo-16 cells)	Prevention
Vitamin E	Plant nuts, plant-based oils, soybeans, wheat germ	Vitamin E prevents the peroxidation of membrane lipids and reduces UVB-induced damage [144].	In vivo (C3H/HeN mice; humans)	Prevention
Vitamin D	Fish, mushrooms	Vitamin D regulates cancer cell proliferation, apoptosis, differentiation, and angiogenesis [142].	In vivo (humans)	prevention
Cryptolepine	*Cryptolepis sanguinolenta*	Cryptolepine expands phosphorilation of ATM/ATR, BRCA1, Chk1/Chk2, and γH2AX, activates the p53 signaling pathway, and promotes apoptosis. It also has an anti-proliferative effect and downregulates cyclin-dependent kinases, cyclin A, cyclin E, cyclin A, cyclin E kinases, and cyclin D1 [155].	In vitro (SCC-13, A431, HaCaT, NHEK cells)	Treatment
Lycoramine Tazettine	Amaryllidaceae	Lycoramine and tazettine, which protected human keratinocytes from UVB-induced production of ROS and IL-6 [154].	In vitro (HaCaT cells)	Prevention
Glycyrrhizic acid	Glycyrrhiza glabra	Glycyrrhizic acid provides protection to skin cells against UVB radiation, a key contributor to NMSC. It reduces cell death and DNA damage, exhibits antioxidant properties, affects autophagy processes, downregulates DNA damage marker proteins, and stabilizes the AKT/PTEN axis disrupted by UVB radiation. Its protective effects are influenced by autophagy regulators [169].	In vitro (human dermal fibroblasts)	Prevention
Betulin, Betulinic Acid, Betulin esters	Betula pendula	Newly modified betulin-originated esters have significant therapeutic potential for actinic keratosis [175].	In vitro (HaCaT cells)	Treatment
		Betulin reduces skin lesions and irritation, notably decreasing erythema, and inhibits the initiation and promotion of skin tumors [176].	In vivo (Balb/c mice)	Prevention
Sulforaphane	Broccoli, broccoli sprouts	SFN reactivates Nrf2, a transcription factor for antioxidant enzymes, by downregulating DNA methyltransferases (DNMTs) and HDACs in JB6 mouse skin epidermal cells exposed to TPA, thus suppressing TPA-induced malignant transformation [191].	In vitro (JB6 P+)	Prevention
		Through the involvement of the Nrf2-dependent mechanism, topical application of SFN on mouse skin results in increased glutathione (GSH) and glutathione S-transferase 4 (GST4) synthesis, which inhibits skin mutagenesis [192].	In vivo (C57BL/6 and Big Blue mice)	Prevention
		The administration of SFN diminishes the levels of PRMT5 and MEP50, thereby causing the formation of H4R3me2s. This phenomenon correlates with decreased cellular proliferation, invasion, and migration of SCC [193].	In vitro (SCC-13, A431, HaCaT cells)	Treatment
Biotin-tagged SFN analog (Biotin-ITC)	Broccoli, broccoli sprouts	The application of a biotin-tagged SFN analog (Biotin-ITC) showed that SFN, through the inhibition of TG2, partially inhibits its binding to GTP, which is crucial for maintaining the aggressive SCC phenotype [194].	In vitro (SCC-13, HaCaT cells)	Treatment
Sulforaphane and cisplatin	Broccoli, broccoli sprouts	Combined therapy of SFN and cisplatin for SCC occurred to suppress tumor formation and reduce the population of cancer stem cells within the tumor [195].	In vitro (SCC-13 cells)	Treatment
AEA	Endogenous	AEA induces ER stress-induced apoptosis in NMSC cells through a receptor-independent mechanism mediated by oxidative stress [202].	In vitro (murine squamous carcinoma cell line JWF2)	Treatment
		AEA is selectively toxic in tumor cells that overexpress COX-2 [201]	In vitro (murine squamous carcinoma cell line JWF2)	Treatment
Lycopene	Tomatoes	Lycopene reduces both the incidence and multiplicity of cutaneous tumors, as well as inhibiting the tumorigenesis of normal cutaneous cells during the promotion phase [208].	In vitro (JB6 P+ cells)	Prevention
Astaxanthin (ASX) Fucoxanthin (FX)	ASX: *Haematococcus pluvialis* FX: Phaeophyceae	FX and ASX inhibit the TPA-induced transformation of mouse skin JB6 P+ cells [213].	In vitro (murine skin JB6 P+ cells)	Prevention
Astaxanthin (ASX)	*Haematococcus pluvialis*	Prior exposure of ASX in human keratinocytes before UVB exposure can inhibit DNA damage [216].	In vitro (HaCaT cells)	Prevention
		The topical application of ASX in mice can protect against UVB-induced DNA damage [215].	In vivo (Wistar mice)	Prevention
Astaxanthin Monesters (AXME) and Diesters (AXDE)	*Haematococcus pluvialis*	AXME and AXDE exhibit a more significant reduction in DMBA-induced tumor incidences compared with ASX alone [217]	In vivo (albino Wistar rats model)	Prevention
C2 Ceramide	Endogenous	C2 ceramide induces apoptosis in human SCC cells (HSC-I)—it is confirmed by dose-dependent toxicity and typical morphological changes in intrinsic apoptosis, such as chromatin condensation, internucleosomal DNA fragmentation, and nuclear fragmentation [223].	In vitro (HSC-I)	Treatment

## Data Availability

Not applicable.
